# The effect of ageing on skeletal muscle as assessed by quantitative MR imaging: an association with frailty and muscle strength

**DOI:** 10.1007/s40520-020-01530-2

**Published:** 2020-03-20

**Authors:** M. Farrow, J. Biglands, S. F. Tanner, A. Clegg, L. Brown, E. M. A. Hensor, P. O’Connor, P. Emery, A. L. Tan

**Affiliations:** 1 Leeds Institute of Rheumatic and Musculoskeletal Medicine, Chapel Allerton Hospital, University of Leeds, Leeds, UK; 2 NIHR Leeds Biomedical Research Centre, Leeds Teaching Hospitals NHS Trust, Leeds, UK; 3 School of Pharmacy and Medical Sciences, University of Bradford, Bradford, UK; 4 Medical Physics and Engineering, Leeds Teaching Hospitals NHS Trust, Leeds, UK; 5 Academic Unit of Ageing and Stroke Research, University of Leeds, Bradford Institute for Health Research, Bradford Teaching Hospitals NHS Foundation Trust, Bradford, UK; 6 Academic Unit of Ageing and Stroke Research, Bradford Institute for Health Research, Bradford Teaching Hospitals NHS Foundation Trust, Bradford, UK

**Keywords:** Muscle, Frailty, T2, MRI, Sarcopenia, Ageing

## Abstract

**Background:**

Skeletal muscles undergo changes with ageing which can cause sarcopenia that can result in frailty. Quantitative MRI may detect the muscle-deficit component of frailty which could help improve the understanding of ageing muscles.

**Aims:**

To investigate whether quantitative MRI measures of T2, fat fraction (FF), diffusion tensor imaging and muscle volume can detect differences within the muscles between three age groups, and to assess how these measures compare with frailty index, gait speed and muscle power.

**Methods:**

18 ‘young’ (18–30 years), 18 ‘middle-aged’ (31–68 years) and 18 ‘older’ (> 69 years) healthy participants were recruited. Participants had an MRI of their dominant thigh. Knee extension and flexion power and handgrip strength were measured. Frailty (English Longitudinal Study of Ageing frailty index) and gait speed were measured in the older participants.

**Results:**

Young participants had a lower muscle MRI T2, FF and mean diffusivity than middle-aged and older participants; middle-aged participants had lower values than older participants. Young participants had greater muscle flexion and extension power, muscle volume and stronger hand grip than middle-aged and older participants; middle-aged participants had greater values than the older participants. Quantitative MRI measurements correlated with frailty index, gait speed, grip strength and muscle power.

**Discussion:**

Quantitative MRI and strength measurements can detect muscle differences due to ageing. Older participants had raised T2, FF and mean diffusivity and lower muscle volume, grip strength and muscle power.

**Conclusions:**

Quantitative MRI measurements correlate with frailty and muscle function and could be used for identifying differences across age groups within muscle.

## Introduction

The increasing proportion of older people in the population (461 million above 65 in 2004 to an estimated 2 billion by 2050 [1]) has significant implications for the planning and delivery of health and social care. Muscle health deteriorates with age, resulting in sarcopenia, reduced muscle mass and strength [2]. It can increase the risk of serious injury from sudden falls and subsequent fractures, especially if the thigh muscles are compromised [3].

The criteria for sarcopenia include low muscle mass, low physical function (such as gait speed) and low muscle strength (such as grip strength). Alongside decreased muscle mass, there is an impairment in muscle quality associated with ageing [4]. Possible explanations for decreases in muscle quality include infiltration of fat into muscle or myosteatosis [3], infiltration of collagen and other non-contractile tissue into muscle [5], progressive atrophy and loss of individual muscle fibres [6], including a decrease in the proportion of fast twitch type II muscle fibres [7], which is associated with a decrease in physical performance [8].

Sarcopenia occurs in up to 13% of those aged above 60 years [9] and is known to cause frailty [10]. Frailty develops because of age-related declines in physiological systems, including muscles, which collectively result in vulnerability to sudden health status changes triggered by minor events, such as a change in medication or minor infection [11]. Up to 50% of people older than 85 years are estimated to be frail, and these people have a substantially increased risk of falls, disability and lower quality of life [12]. Reducing the prevalence or the severity of frailty is likely to have large benefits for individuals and society [13].

Magnetic resonance imaging (MRI) has the potential to enable better targeting of interventions based on well-defined quantitative measures of sub-clinical muscle differences that are associated with frailty [14]. Quantitative MRI (qMRI) measurements have shown promising results for evaluating skeletal muscles by overcoming limitations in visual assessment based on gross morphologic and signal intensity changes. Transverse relaxation time (T2) measurements, fat fraction (FF), diffusion tensor imaging (DTI) and muscle volume have all shown sensitivity to muscle differences in response to disease, and have excellent inter-scan variability [15]. T2 measurements are sensitive to fluid levels within the muscle and can identify muscle oedema [16]. Metabolic fat changes is associated with bone changes such as osteoarthritis and osteoporosis that tend to occur in older people [17]; MR-based FF measurements are able to quantify the degree of steatosis in organs [18], and specifically myosteatosis in the muscle which is regarded as a measure of muscle quality [19]. DTI is sensitive to muscle tissue microstructure, and may be useful in the assessment of changes of muscle fibres [20] and have been shown to be sensitive to differences due to age [21]. MRI-based muscle volume measurements can be used to quantify the loss of muscle mass. Muscle mass has been shown to be associated with fractures [22], and loss of muscle mass is a main diagnostic criterion for sarcopenia [23].

Whilst these measures have been demonstrated to be sensitive to age-related differences in muscle, they have not been compared with formal measurements of frailty or with quantitative muscle power measurements. These relationships need to be understood if qMRI measurements are to be used clinically as diagnostic or monitoring biomarkers. Therefore, the aim of this study was to investigate whether qMRI techniques are sufficiently sensitive to detect differences in muscle properties between young, middle-aged and older participants and to show how qMRI parameters relate to muscle function and frailty.

## Methods

### Study design

This prospective, cross-sectional study was conducted at Leeds Teaching Hospitals NHS Trust (LTHT). It was approved by the local research ethics committee (17/EM/0079) and all participants provided written informed consent. Participants were recruited between May 2017 and December 2018 into three sex-matched age groups: ‘young’ (18–30 years), ‘middle-aged’ (31–68 years), and ‘older’ (≥ 69 years). The age classifications for the young and older participant groups were chosen based on the European MyoAge study [24]. Sample size was based on published guidelines recommending between 12 and 30 participants per group to estimate parameters for powering future trials [25]. The older participants included participants from a longitudinal research cohort [The Community Ageing Research 75 + (CARE-75 +) Study] (Trial registration number ISRCTN16588124) [26]. The English Longitudinal Study of Ageing (ELSA) frailty index (FI) scores (0–10 = very fit, 11–14 = well, 15–24 = vulnerable, 25 + frail [27]) were obtained for the older participants to investigate correlation between MRI and muscle function with FI.

Inclusion criteria included (1) being asymptomatic of muscle disease, (2) no previous history of musculoskeletal disorders, (3) no corticosteroid treatment within the past 3 years with doses > 5 mg/day, (4) no HMG-CoA reductase inhibitors for the past 3 years. These criteria were ascertained by self-reporting from the participant.

Exclusion criteria for participants were age < 18 years, contraindications to MRI, previous history of muscle disorder, spinal disease and neuropathy, an ELSA frailty index score of ≤ 14 to ensure participants could carry out the study. Osteoarthritis was not an exclusion criterion due to its high prevalence in the older population.

### Magnetic resonance imaging measurements

MR data were acquired using a MAGNETOM Verio 3T MR scanner (Siemens Healthcare, Erlangen, Germany). The imaging protocol has been previously described [15]. Images of the dominant thigh (the right if the participant was unsure) were acquired using two small four-channel flex coils.

All quantitative images were aligned to each other and acquired with the same field of view to enable cross-propagation of regions of interest (ROI)s. For fat quantitation, a 40-slice, volume-interpolated breath-hold examination (VIBE), 2-point Dixon sequence was used [15]. Diffusion-weighted images were acquired using a STimulated Echo Acquisition Mode (STEAM) prototype sequence, with an echo-planar imaging (EPI) readout [28] and spectral adiabatic inversion recovery (SPAIR) fat suppression [15].

For T2 measurements, axial images were obtained using a T2-weighted, multi-echo, spin-echo (MESE) sequence with SPAIR fat suppression with an echo train length 16, and echo times (TE) of 9.6, 19.2, 28.8, 38.4, 48.0, 57.6, 67.2, 76.8, 86.4, 96.0, 105.6, 115.2, 124.8, 134.4, 144.0, 153.6 ms, repetition time (TR) of 1500 ms, slice thickness 5 mm, matrix 256 × 256, number of averages = 1, with a field of view of 300 × 300 mm.

Regions of interest (ROIs) were contoured using Osirix imaging software (version 4.0; open-source DICOM viewer, https://osirix-viewer.com). Regions depicting the individual hamstring and quadriceps muscles were drawn on the middle slice of the in-phase VIBE Dixon volume for each participant, avoiding fascial tissue and subcutaneous fat. ROIs were copied to the corresponding diffusion parameter maps, accounting for differences in image resolution, and the mean value within each ROI was measured. The quantitative MRI slice analysed corresponded to the central slice (slice 20) of the VIBE Dixon muscle volume.

Fat fraction values were calculated from the fat and water images generated from the VIBE Dixon images for each ROI. To calculate T2 values the signal intensity versus echo time decay curves from each ROI were fitted using a mono-exponential decay function. To reduce the effect of additional signal from stimulated echoes, the signal from the earliest time point was excluded from the fit [29].

Muscle volume estimates were obtained using a semi-automated algorithm that used fat fraction maps generated from the VIBE Dixon volume data. The algorithm only assigned a voxel as being muscle provided it did not correspond to regions of bone and had a fat fraction of less than 50%. Muscle from the contralateral leg was excluded using a bounding box. Bone was excluded using a 3D-connected components algorithm (bwconncomp, MATLAB) from a seed point manually placed within the bone on the central slice of the VIBE Dixon volume. Finally, a mask defining the muscle was obtained by thresholding, using a fat fraction threshold of < 50% for muscle. This threshold has been previously used in muscle volume measurements in the erector spinae muscles [30]. Muscle masks were only defined between slice 5 and 35 of the 40-slice volume to avoid errors due to signal drop-off at the outer extremities of the receive coil. The volume was defined as the number of voxels in the muscle mask multiplied by the voxel size, multiplied by the slice width.

### Muscle strength assessments

Knee extension and flexion isokinetic assessment of the dominant thigh were performed following MRI at a controlled room temperature of 20 °C using an isokinetic biodex system 4 muscle testing and rehabilitation isokinetic dynamometer (IPRS Mediquipe Limited, UK). Participants were instructed to refrain from strenuous physical activity for 24 h prior to assessment. After a standardised warm-up, participants were positioned according to the manufacturer’s instructions. Gravitational correction was performed at 180°. Isokinetic knee extension–flexion (concentric–concentric) at 60°/s was used to collect data. Participants performed three maximum effort repetitions for three sets, separated by a 30 s rest interval. Standardized verbal stimuli were provided throughout the evaluation. Power (Watts) was the assessed variable. Handgrip strength was also measured using a Jamar plus isokinetic dynamometer. Participants had their grip strength measured in their dominant hand for three sets and the mean value was recorded. In older people only, gait speed was measured by conducting a 4 m walk test [23].

### Statistical analyses

Offline image analysis was performed using MATLAB software (R2018b, Mathworks, Nattick, MA, USA). Statistical analyses were performed using SPSS (IBM SPSS Statistics for Windows, Version 25.0. Armonk, NY: IBM Corp). One-Way ANOVA with a Bonferroni post hoc analysis was used to test for significant differences in quantitative MR, handgrip strength and muscle power measurements between all groups.

Spearman’s rank correlation was used to measure correlation. *r*_s_ Values ≥ 0.3 were considered as indicative of potential correlation. Correlations between participants with handgrip and frailty were only calculated in older participants who had undergone an ELSA frailty index assessment.

## Results

18 young (mean age 26 ± 8), 18 middle-aged (mean age 49 ± 19), and 18 older (mean age 79 ± 5 and mean ELSA frailty index score 10 ± 5) participants took part in this study. None of the older participants had sarcopenia as determined by the EWGSOP sarcopenia classification criteria [23]. Each group consisted of nine males and nine females. There were differences in quantitative MRI and muscle strength between all groups. Descriptive data for quantitative MRI and muscle power/volume measurements are shown in Tables 1and 2.

**Table 1:** Quantitative MRI measurements with post hoc Bonferroni *t* test to determine significance between all 3 groups

	T2 (ms)			Fat fraction (%)			Mean diffusivity (× 10^−3^ mm^2^ s)			Fractional anisotropy		
	Mean (SD)	95% CI	*p* value	Mean (SD)	95% CI	*p* value	Mean (SD)	95% CI	*p* value	Mean (SD)	95% CI	*p* value
Hamstrings												
Young (*n* = 18)	39.3 (1.8)	38.5, 40.3	< 0.001	3.4 (1.6)	2.7, 4.3	< 0.001	1.26 (0.1)	1.23, 1.29	< 0.001	0.42 (0.04)	0.40, 0.44	0.2
Middle-aged (*n* = 18)	40.8 (1.4)	40.1, 41.6		5.6 (2.2)	4.4, 6.7		1.34 (0.1)	1.28, 1.39		0.40 (0.1)	0.39, 0.41	
Older (*n* = 18)	42.9 (2.9)	41.4, 44.4		9.5 (3.6)	7.6, 11.3		1.40 (0.1)	1.34, 1.45		0.39 (0.04)	0.35, 0.41	
Quadriceps												
Young (*n* = 18)	40.6 (1.4)	40, 41	< 0.001	2.2 (0.8)	1.9, 2.6	< 0.001	1.30 (0.1)	1.26, 1.33	< 0.001	0.35 (0.05)	0.30, 0.40	0.7
Middle-aged (*n* = 18)	42.8 (2.1)	41.9, 43.9		3.2 (2.3)	2.4, 4.8		1.35 (0.1)	1.29, 1.33		0.34 (0.05)	0.30, 0.40	
Older (*n* = 18)	45.0 (2.4)	43.8, 46.2		6.4 (1.9)	5.2, 7.1		1.41 (0.1)	1.29, 1.41		0.33 (0.06)	1.3, 0.4

**Table 2:** Muscle volume, Knee extension and flexion and handgrip strength measurements

	Muscle volume (cm^3^)			Flexion power (w)			Extension power (w)			Handgrip strength (kg)		
	Mean (SD)	95% CI	*p* value	Mean (SD)	95% CI	*p* value	Mean (SD)	95% CI	*p* value	Mean (SD)	95% CI	*p* value
Young (*n* = 18)	1563 (412)	1357.6, 1768.3	0.002	45 (22.0)	34, 55	< 0.001	84 (43.2)	63, 106	< 0.001	36.8 (10.7)	31.5, 42.2	< 0.001
Middle-aged (*n* = 18)	1365 (362)	1195.6, 1534.8		33 (11.1)	26, 37		51 (20.4)	39, 60		31.5 (5.1)	28.9, 34.2	
Older (*n* = 18)	1151 (327)	988.7, 1314.4		18 (9.9)	12, 22		28 (18.3)	18, 37		23.1 (9.7)	18.1, 28.1	

### Muscle T2

**Fig. 1:**
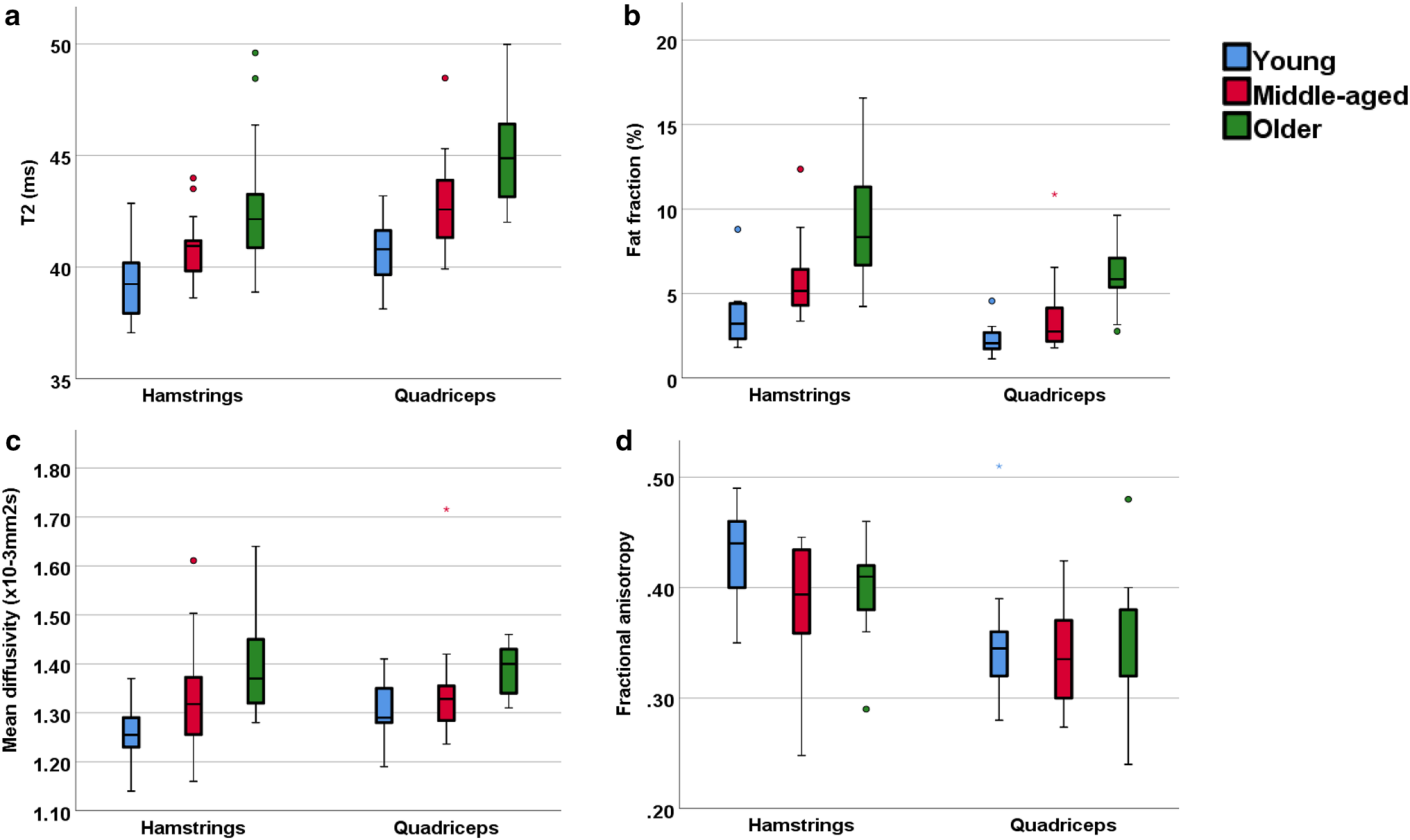
Quantitative MRI measurements of young, middle-aged and older participant groups. **a** T2, **b** fat fraction, **c** mean diffusivity, **d** fractional anisotropy

T2 increased with age (Table 1). Within the hamstrings, differences between young and old, young and middle-aged, middle-aged and older participants were 3.6 ms (95% CI 1.8, 5.2; *p* < 0.001), 1.5 ms (95% CI 0.4, 2.7; *p* = 0.01) and 2.1 ms (95% CI 0.3, 3.7; *p* = 0.02), respectively. Within the quadriceps these differences were 4.4 ms (95% CI 2.8, 5.9; *p* < 0.001), 2.2 ms (95% CI 1, 3.4; *p* = 0.001), and 2.2 ms (95% CI 0.6, 3.7; *p* = 0.005), respectively (Fig. 1).

### Muscle fat fraction

Fat fraction increased within each age group increment (Table 1). Within the hamstrings, differences between young and old, young and middle-aged, middle-aged and older participants were 6.1% (95% CI 4.0, 8.2; *p* < 0.001), 2.2% (95% CI 0.8, 3; *p* = 0.003) and 3.9% (95% CI 2, 6; *p* < 0.001), respectively. Within the quadriceps, the differences were 4.2% (95% CI 3, 5; *p* < 0.001) 1.0% (95% CI 0.2, 3; *p* = 0.02) and 3.2% (95% CI 1, 4; *p* < 0.001), respectively (Fig. 1).

### Muscle diffusion tensor imaging

Mean diffusivity (MD) increased with age (Table 1). Within the hamstrings, differences between young and old, young and middle-aged, middle-aged and older participants for MD were 0.14 × 10^−3^ mm^2^ s (95% CI 0.06, 0.21; *p* < 0.001), 0.08 × 10^−3^ mm^2^ s (95% CI 0.01, 0.1; *p* = 0.01) and 0.06 × 10^−3^ mm^2^ s (95% CI 0.11, 0.13; *p* = 0.1), respectively. Within the quadriceps, the differences were 0.11 × 10^−3^ mm^2^ s (95% CI 0.03, 0.16; *p* = 0.002), 0.05 × 10^−3^ mm^2^ s (95% CI 0.01, 0.1; *p* = 0.1) and 0.06 × 10^−3^ mm^2^ s (95% CI 0.03, 0.11; *p* = 0.2), respectively (Fig. 1), demonstrating higher MD in older participant groups.

There were no substantial differences in fractional anisotropy (FA) between age groups (Table 1). Within the hamstrings, differences between young and old, young and middle-aged, middle-aged and older participants for were 0.03 (95% CI 0.01, 0.06; *p* = 0.3), 0.02 (95% CI 0.01, 0.06; *p* = 0.3) and 0.01 (95% CI 0.01, 0.02; *p* = 0.9), respectively. Within the quadriceps, these were 0.02 (95% CI 0.01, 0.03; *p* = 0.9), 0.01 (95% CI 0.01, 0.02; *p* = 0.5), and 0.01 (95% CI 0.01, 0.03; *p* = 0.9), respectively (Fig. 1).

### Muscle volume

Muscle volume decreased with age (Table 2). There were differences in muscle volume between young and old, young and middle-aged, middle-aged and older participants of 412cm^3^ (95% CI 106, 690; *p* = 0.006), 198cm^3^ (95% CI 0, 500; *p* = 0.1) and 214 cm^3^ (95% CI 62, 493; *p* = 0.1), respectively.

### Muscle power and grip strength

Muscle power measurements for knee flexion were related to the hamstrings, and for knee extension to the quadriceps, which are the primary muscles for the forms of movement, respectively. Muscle power and grip strength decreased between each age group increment (Table 2). There was a difference in hamstring flexion power between young and old, young and middle-aged, middle-aged and older participants of 27 W (95% CI 15, 39; *p* < 0.001), 12 W (95% CI 1, 25; *p* = 0.03) and 15 W (95% CI 2.2, 27.4; *p* = 0.01), respectively.

Within the quadriceps, there was a difference in extension power between young and old, young and middle-aged, middle-aged and older participants of 56 W (95% CI 33, 79; *p* < 0.001), 33 W (95% CI 11, 58; *p* = 0.005) and 23 W (95% CI 1, 45; *p* = 0.07), respectively, demonstrating lower muscle power in the older groups.

There was a difference in handgrip strength between young and old, young and middle-aged, middle-aged and older participants of 13.7 kg (95% CI 6.6, 20.8; *p* < 0.001), 5.3 kg (95% CI 1, 11; *p* = 0.07) and 8.4 kg (95% CI 3.1, 13.9; *p* = 0.003), respectively.

### MRI and muscle function in all participants

**Fig. 2:**
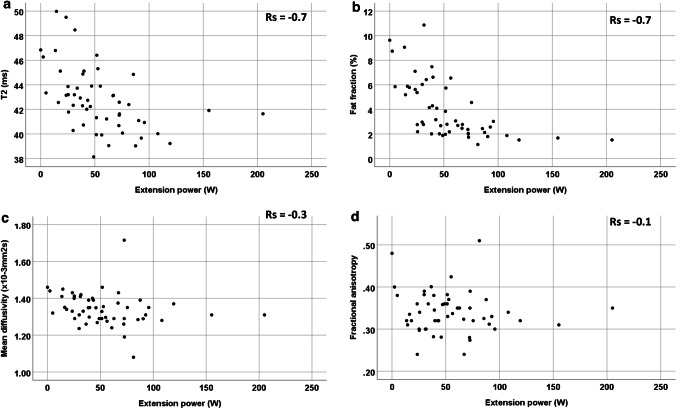
Quantitative MRI of the quadriceps and correlation versus extension power for all participants (young, middle-aged and older participants combined as one). **a** T2, **b** fat fraction, **c** mean diffusivity, **d** fractional anisotropy

**Fig. 3:**
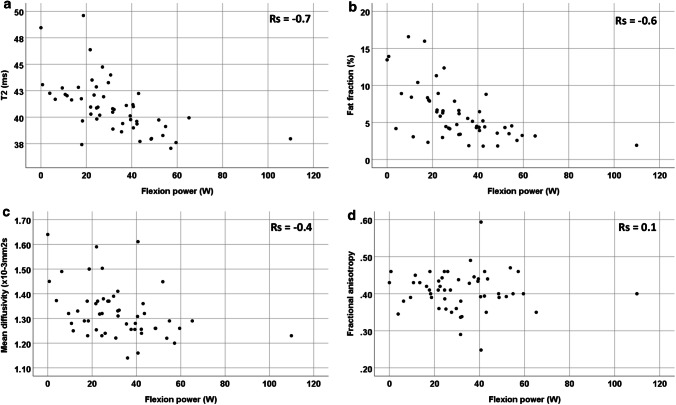
Quantitative MRI of the hamstrings and correlation versus flexion power for all participants (young, middle-aged and older participants combined as one). **a** T2, **b** fat fraction, **c** mean diffusivity, **d** fractional anisotropy

Considering the entire 54 participant dataset: (Figs. 2, 3), T2 correlated with flexion (*r*_s_ = −0.7; *p* < 0.001), extension (*r*_s_ = −0.7; *p* < 0.001) and handgrip strength (*r*_s_ = −0.6; *p* < 0.001). FF correlated with flexion (*r*_s_ = −0.6; *p* < 0.001), extension (*r*_s_ = −0.7; *p* < 0.001) and handgrip strength (*r*_s_ = −0.6; *p* < 0.001). MD correlated with flexion (*r*_s_ = −0.4; *p* = 0.04), extension (*r*_s_ = −0.3; *p* = 0.05) but did not correlate with handgrip strength (*r*_s_ = −0.1; *p* = 0.9). FA did not correlate with any of the muscle functions: FA with flexion power (*r*_s_ = 0.1; *p* = 0.9), extension power (*r*_s_ = −0.1; *p* = 0.1), and handgrip strength (*r*_s_ = 0.01; *p* = −0.9). Handgrip strength was also found to be correlated with flexion power (*r*_s_ = 0.7; *p* < 0.001) and extension power (*r*_s_ = 0.7; *p* < 0.001).

### MRI, muscle function and frailty index in older participants

As only participants in the older group were scored with the ELSA frailty index (FI) [31], the association between muscle function parameters with the ELSA frailty index could only be assessed in this group.

**Fig. 4:**
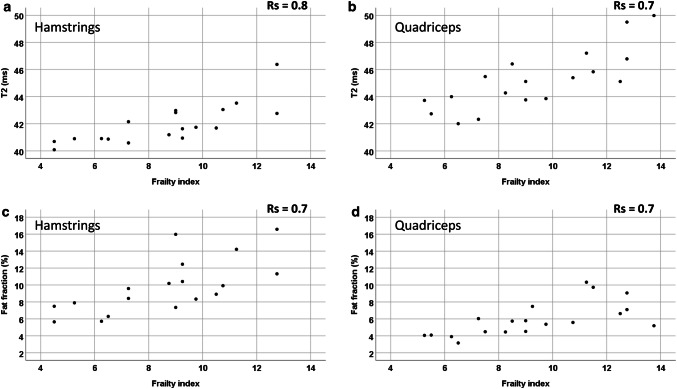
Quantitative T2 and FF MRI and frailty index correlation of older participants in the hamstrings and quadriceps. **a** T2 hamstrings, **b** T2 quadriceps, **c** fat fraction hamstrings, **d** fat fraction quadriceps

Quantitative MRI and muscle volume and knee flexion and extension correlated with FI and gait speed in older participants (Fig. 4).

In the hamstrings, T2 correlated with FI *r*_s_ = 0.8; *p* < 0.001, gait speed *r*_s_ = −0.4, *p* = 0.05, knee flexion *r*_s_ = −0.7, *p* = 0.01, and in the quadriceps with FI *r*_s_ = 0.7, *p* < 0.001, gait speed *r*_s_ = −0.5; *p* = 0.007, knee extension *r*_s_ = −0.6; *p* < 0.001.

In the hamstrings FF correlated with FI *r*_s_ = 0.7, *p* < 0.001, gait speed *r*_s_ = −0.4; *p* = 0.02, knee flexion *r*_s_ = −0.6; *p* = 0.001 and in the quadriceps with FI *r*_s_ = 0.7; *p* < 0.001, gait speed *r*_s_ = −0.6, *p* = 0.001, knee extension *r*_s_ = −0.7; *p* < 0.001.

In the hamstrings MD was weakly correlated with FI *r*_s_ = 0.3; *p* = 0.2, gait speed *r*_s_ = −0.3, *p* = 0.1, knee flexion *r*_s_ = −0.4, *p* = 0.004 and in the quadriceps with FI *r*_s_ = 0.4, *p* = 0.1, gait speed *r*_s_ = −0.3, *p* = 0.2, knee extension *r*_s_ = −0.4; *p* = 0.007.

**Fig. 5:**
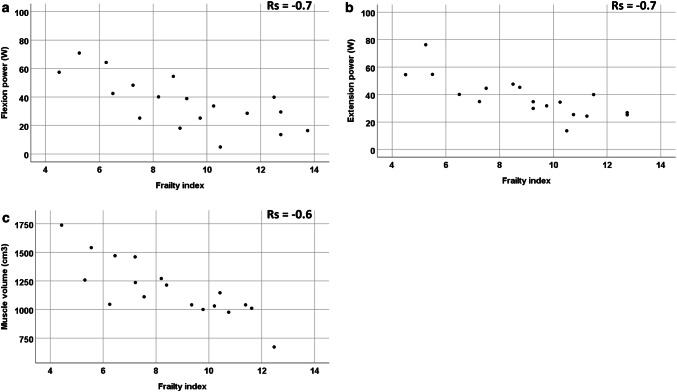
Muscle volume and muscle power versus frailty index correlation of older participants. **a** Knee flexion power, **b** knee extension power, **c** muscle volume

Muscle volume (Fig. 5) correlated with FI *r*_s_ = −0.6; *p* < 0.001, gait speed *r*_s_ = 0.6; *p* = 0.01, knee flexion *r*_s_ = 0.6; *p* < 0.001, knee extension *r*_s_ = 0.6; *p* < 0.001.

Knee flexion (Fig. 5) correlated with FI (*r*_s_ = −0.7, *p* = 0.002), gait speed (*r*_s_ = −0.4, *p* = 0.05) and grip strength (*r*_s_ = 0.7; *p* < 0.001). Knee extension (Fig. 5) correlated with FI (*r*_s_ = −0.7; *p* = 0.001), gait speed (*r*_s_ = 0.5; *p* = 0.01) and grip strength (*r*_s_ = 0.7; *p* < 0.001).

Handgrip strength correlated with frailty index and gait speed (FI *r*_s_ = −0.7, *p* = 0.001, gait speed *r*_s_ = 0.5, *p* = 0.06).

## Discussion

This study has shown that MRI T2, FF, MD, muscle volume differs across age groups. Furthermore, these measurements also correlated with muscle power and strength measurements and with an independent measure of frailty. This suggests that, not only is MRI an independent measure of muscle strength, but also shows potential as a quantitative adjunct assessment of muscle strength and frailty. As current measures of frailty can be subjective or qualitative, MRI-based measures could be the basis of a more robust measure of frailty. Finally, we have shown that grip strength correlates with lower limb function and frailty index and, therefore, has some utility as an indicative measure of overall muscle function status.

The increase of muscle T2 with age agrees with previous work [32]. Ageing is known to be associated with increased systemic inflammatory markers, such as CRP, IL-1RA and IL-6 [33]. There is evidence to suggest that T2 is raised in muscle inflammation [34]. However, this may be due to inadequate fat suppression increasing the T2 values [35]. We found that the FF was higher in the older participants, suggesting that fat is also increased with age, therefore, this effect cannot be ruled out as contributing to the raised T2 in our study.

Muscle volume correlated with muscle power and there were differences in muscle volume between the age groups. As muscle mass is a primary component in the diagnosis of sarcopenia, the more accurate muscle volume measurements available with quantitative MRI may be useful in the diagnosis of sarcopenia, which currently has no accepted diagnostic classification criteria.

Mean diffusivity was greater with age and there were small, non-significant, differences in fractional anisotropy, with lower measurements in older participants. Previous studies reporting the relationship between age and muscle water MD have shown results that differ from those presented here [21]. This discrepancy has arisen due to the differing experimental methods employed to measure diffusion. This is a consequence of our use of a long diffusion time (1000 ms), compared to a spin-echo sequence, that can be utilised when carrying out measurements in muscle using the STEAM technique to allow for increased water diffusion across the width of the muscle fibre. Our observed trend of increasing MD with age agrees with a number of previous studies including that of Sinha et al. [14] who also used DTI to acquire MD. This increase in MD can be interpreted using a model of muscle ageing in which differences in these diffusion metrics occur due to fibre atrophy and a relative increase in fibre cross-sectional asymmetry. Thus, the increase in MD could be due to the increased amount of intramuscular extracellular connective tissue.

Handgrip tests are frequently used as a proxy measure of global muscle strength [36]. Whilst it is not correct to equate forearm strength with muscle parameters of the thigh, handgrip strength is a frequently used assessment of frailty and sarcopenia [72, 80]. As far as the authors are aware, this is the first study to demonstrate an association of handgrip strength with qMRI measurements in the thigh. This validates handgrip strength as a useful indicator of overall muscle strength, although it does not provide information on muscle quality, such as fatty infiltration and inflammation.

Our study is subject to limitations. We acknowledge that this is a pilot study, and therefore, the study has a relatively small sample size. Multi-echo sequences over-estimate T2 due to the formation of stimulated echoes [29], but are commonly used in clinical practice to keep scan times tolerably short for participants. More elegant analysis methods that take the full extended phase graph into account have been used [37], however, these methods are complex and not easily available in clinical settings. The two-point Dixon imaging technique did not correct for T2* effects, eddy currents, noise related bias, or the spectral complexity of fat, although they correlate with confounder-corrected fat quantitation methods and with spectroscopy [38]. Only 6 diffusion directions were used to decrease the scan times, when 12 are recommended to reduce bias between the encoding and underlying tissue, which could have limited the sensitivity of our measurements to differences in diffusion [39]. Our muscle volume measurements did not consider differences in shape and length of thigh muscles between patients, although we attempted to control for these differences by positioning relative to an anatomical reference marker, similar to previous studies [40]. However, work has also been published demonstrating the validity of measuring total muscle volume using one slice as it is frequently reported [41].

In conclusion, ageing is associated with greater MRI T2, FF, mean diffusivity and lower muscle volume, grip strength and muscle power. Quantitative MRI parameters correlated with grip strength, muscle power and the ELSA frailty index. Quantitative MRI measurements have the potential to be useful markers of age and muscle health and could be used in the management of sarcopenia and frailty.
